# Incidence, analysis and risk of out-of-hospital cardiac arrest in individuals with non-ischaemic dilated cardiomyopathy

**DOI:** 10.1093/eschf/xvag207

**Published:** 2026-07-31

**Authors:** Leo Henry Mansell, Peder Emil Warming, Rasmus Bork Dinesen, Rodrigue Garcia, Reza Jabbari, Gunnar Gislason, Bo Gregers Winkel, Jacob Tfelt-Hansen

**Affiliations:** Department of Cardiology, Copenhagen University Hospital—Rigshospitalet, Copenhagen, Denmark; Department of Cardiology, Copenhagen University Hospital—Rigshospitalet, Copenhagen, Denmark; Department of Cardiology, Copenhagen University Hospital—Rigshospitalet, Copenhagen, Denmark; Department of Cardiology, Copenhagen University Hospital—Rigshospitalet, Copenhagen, Denmark; Cardiology Department, University Hospital of Poitiers, Poitiers, France; Centre D'Investigation Clinique 1402, University Hospital of Poitiers, Poitiers, France; Department of Cardiology, Copenhagen University Hospital—Rigshospitalet, Copenhagen, Denmark; Department of Cardiology, Copenhagen University Hospital—Rigshospitalet, Copenhagen, Denmark; Department of Cardiology, Copenhagen University Hospital—Herlev and Gentofte Hospital, Gentofte, Denmark; The Danish Heart Foundation, Copenhagen, Denmark; The Department of Clinical Medicine, University of Copenhagen, Denmark; Department of Cardiology, Copenhagen University Hospital—Rigshospitalet, Copenhagen, Denmark; Department of Cardiology, Copenhagen University Hospital—Rigshospitalet, Copenhagen, Denmark; Department of Forensic Medicine, Faculty of Health and Medical Sciences, Copenhagen, Denmark

**Keywords:** OHCA, DCM, Shockable Rhythm

## Abstract

**Introduction:**

The incidence, risk and resuscitation characteristics of out-of-hospital cardiac arrest (OHCA) in the non-ischaemic dilated cardiomyopathy (NIDCM) population have not been analysed before in an unselected nationwide population, and could provide insight into risk stratification and prevention of sudden cardiac death in this unique heart failure cohort.

**Methods:**

We conducted an observational, register-based study with cohort and nested case-control analyses using Denmark’s healthcare registers between 1 June 2001 to 31 December 2022. DCM was classified as non-ischaemic using validated methods combined with exclusion of other causes of ischaemia or abnormal loading conditions. Incidence rates and hazard ratios were calculated in the general population. Absolute risk was determined using the Aalen-Johansen estimator in an exposure-matched cohort including incident NIDCM patients and matched controls. Resuscitation characteristics were determined in a nested case-control study.

**Results:**

The incident rate of OHCA was approximately 12 times higher in NIDCM patients compared with the general population (incidence rate 532 vs 45 per 100 000 person years). The 5- and 10-year risk of OHCA for male *de novo* NIDCM patients was 2% and 3.4%, respectively (vs 0.6% and 1.1% for non-NIDCM) and 1.4% and 2.1% for female *de novo* NIDCM patients (vs 0.4 and 0.5% for non-NIDCM). NIDCM OHCAs had higher rates of initial shockable rhythm than non-NIDCM OHCAs (48.5% vs 22.3%, *P* < .001) but no significant differences in 30 day and 1-year mortality (79% vs 84% and 82% vs 84, respectively).

**Conclusions:**

The Danish NIDCM population has a significantly increased incidence of OHCA compared to the general population but a comparable mortality despite multiple positive resuscitation characteristics.

## Graphical Abstract

**Graphical Abstract:**
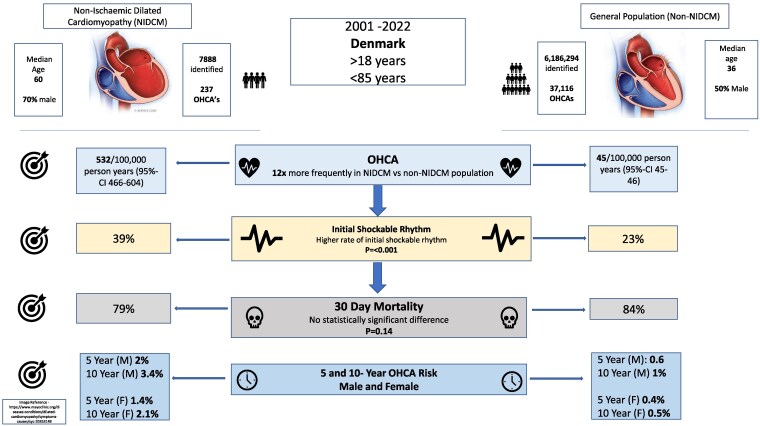
Between 2001 and 2022, OHCA in patients with confirmed NIDCM was compared to OHCA in the general population. The results showed a greater incidence and long term risk of OHCA in the NIDCM, along with a greater frequency of initial shockable rhythm. However, there was no statisitically significant difference in 30 day mortality between the groups.

## Introduction

Non-ischaemic dilated cardiomyopathy (NIDCM) is characterized by progressive, often biventricular dilation and systolic dysfunction in the absence of both coronary artery disease and abnormal loading conditions.^1^ Cases may be sporadic or familial in origin,^2^ and although the majority of cardiac causes of death will be progressive systolic dysfunction, up to 35% of all NIDCM mortalities are thought to be secondary sudden cardiac death (SCD)^3^ due to a ventricular arrhythmia (VA), the majority of which occur in the form of an out-of-hospital cardiac arrest (OHCA).

OHCA and SCD are widely studied and monitored globally,^4,5^ with coronary artery disease (CAD) accounting for the majority of causes.^6^ For NIDCM patients, who have minimal or no burden of CAD, focus remains on identifying, stratifying, and subsequently preventing sudden death. Prior analyses of the characteristics and incidence of OCHA in the Danish population have been published,^7^ providing data across a variety of parameters including location, CPR, and initial shockable rhythm. However, a focused analysis of OHCA in the NIDCM population has not been performed to date on a nationwide scale. Using Denmark’s unique health registries, our objective was to investigate the incidence and long-term risk, along with a selection of resuscitation characteristics, of OHCA in the Danish NIDCM population.

## Methods

### Study design and data sources

This was a nationwide, observational, register-based study that integrated both a cohort and nested case-control study. The study analysed pre-existing population-level data across multiple national Danish registers,^8^ was designed in line with the STROBE guidelines and meets all minimum standards of CODE-EHR Framework.

Denmark boasts several healthcare registers which can provide large quantities of demographic and clinical data for research purposes. Data can be cross-linked on an individual level due to the unique civil registration number all citizens of Denmark are assigned at either birth or first-time immigration. Each time an individual interacts with the Danish health service and/or acquires a new diagnosis, the corresponding diagnostic code from the International Classification of Diseases 10th Revision (ICD-10) is linked to their unique civil registration number and entered into the relevant national register. This allows researchers to work with data on a population-wide scale. We used the following registers to obtain the relevant data: (i) The Danish National Population Register to identify the entire Danish population,^9^ (ii) The Danish National Patient Register to obtain information on all hospital admissions, ambulatory visits, ICD-10 diagnosis codes, and procedures (classified according to Nordic Classification of Surgical Procedures),^10^ (iii) The Danish Cause of Death Register to obtain information on vital status and cause of death^11^ (iv) The Danish Cardiac Arrest Register to obtain information on all OHCA’s from 1 June 2001 to 31 December 2022.^12^

### Study population

The initial study population included all living individuals between 18 and 85 years of age, residing in Denmark between 1 June 2001 and 31 December 2022. All individuals with a diagnosis of NIDCM were first identified using ICD-10 code DI420, previously validated to an accuracy of 82%.^13^ We then further refined this initial NIDCM population by excluding any patients with exposure to an underlying ‘explainable disease’: hypertension, chronic kidney disease, valve disease, and ischaemic heart disease, (i.e. a diagnosis that may cause abnormal loading conditions or myocardial ischaemia) before the study started.^14^ ‘Explainable diseases’ were identified using their corresponding ICD-8 or −10 codes (a full list of ICD-8 and 10 codes used for defining explainable diseases is provided in the Supplementary Table S1). Hypertension was determined by proxy via medication using a previously validated method shown to be more accurate in this circumstance.^15^ Hypertension was confirmed present if an individual had claimed at least two classes of antihypertensive medication prescriptions within 180 days prior to the index date (a full list of ATC-codes used to define hypertension is provided in Supplementary Table S2). Individuals were regarded as having a comorbidity at the first occurrence of the diagnosis code.

Two distinct subpopulations were sampled from the study population—one for the cohort study and the other for the nested case-control study.

### Cohort study

All patients with a first-time diagnosis of NIDCM (exposure) between 1 June 2001 and 31 December 2022 were included for the cohort study and used to determine the long-term risk of OHCA (*de novo* NIDCM). All *de novo* NIDCM were matched at the time of diagnosis with individuals without a diagnosis of NIDCM (unexposed) from the source population. They were matched on sex, birth year, as well as comorbidities (stroke, atrial fibrillation, chronic obstructive pulmonary disease (COPD), diabetes, cancer) and usage of statins (Supplementary Table S3) in a matching ratio of 1:4. Patients with *de novo* NIDCM were excluded if they had an inadequate number of matches to maintain the matching ratio.

### Nested case-control study

All patients with a diagnosis of NIDCM who experienced an OHCA during the observation period (NIDCM OHCA)—1 June 2001–31 December 2022—were included for the nested case-control study. NIDCM OHCAs were matched at the time of event with patients who had experienced an OHCA but had no diagnosis of NIDCM (non-NIDCM OHCA). They were matched in a 1:4 ratio on sex, exact birth year, and year of OHCA.

### Statistics

Individuals were at risk from age 18, 1 June 2001, or immigration to Denmark (whichever occurred last) until OHCA, death, development of explainable disease, age 85, emigration from Denmark, or 31 December 2022 (whichever occurred first). For the Cox models, NIDCM diagnosis, as well as other comorbidities, were treated as time-dependent exposures. As such, individuals developing NIDCM during the study period contributed risk time to the non-exposed group until NIDCM diagnosis. Median follow-up is reported using the reverse Kaplan–Meier method.^16^ The incidence rate and incidence rate ratio (IRR) of OHCA were calculated along with Poisson confidence intervals. Multivariable adjusted Cox proportional hazards models were used with age as the underlying timescale and presented as hazard ratios (HR). Estimates were adjusted for sex, diagnosis of stroke, atrial fibrillation, chronic obstructive pulmonary disease, peripheral artery disease, diabetes, cancer, and statin use. The cumulative incidences are presented using the Aalen-Johansen estimator, and matching was performed using exposure density sampling.^17^

### Ethics

Danish register-based studies performed for the purpose of scientific research do not require ethical committee approval or patient consent. Access to data was granted by the data responsible institute in the Capital Region of Denmark (approval number *P*-2019-191). Danish legislation prohibits sharing of register data and low-frequency numbers (*n* ≤ 3) are not to be reported and are subject to censoring.

## Results

### Study population and baseline characteristics

**Figure 1:**
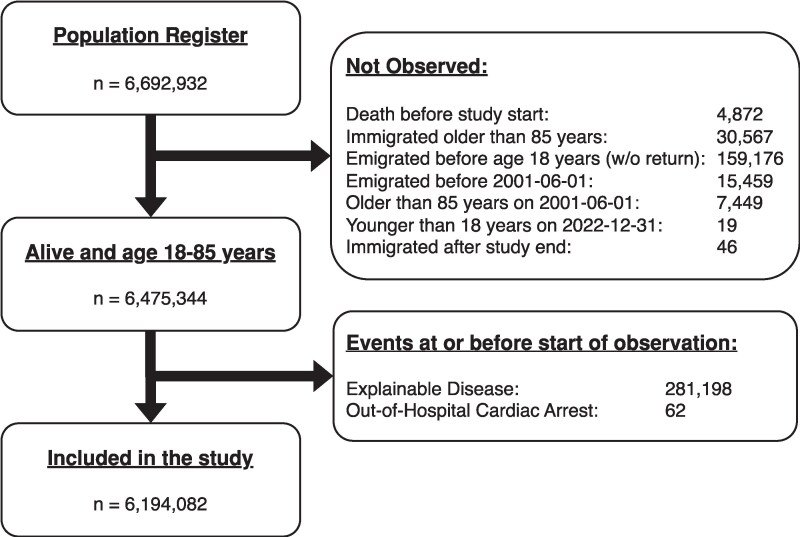
Flowchart of study population identification. Flowchart demonstrating the creation of the study population, defined as all living individuals between 18 and 85 years of age, residing in Denmark between 1 June 2001 and 31 December 2022. The non-ischaemic dilated cardiomyopathy (NIDCM) cohort was identified using ICD-10 code (DI420), further refined by excluding patients with any exposure to an underlying ‘explainable disease’: hypertension, chronic kidney disease, valve disease, and ischaemic heart disease

We initially identified 6 475 344 individuals who were alive, residing in Denmark, and aged 18–85 years during the study period. After excluding individuals with OHCA occurring before age 18 and those with a pre-existing explainable condition at study entry, a total of 6 194 082 individuals were eligible for inclusion in the analysis (*Figure 1*).

A total of 1047 patients had a diagnosis of NIDCM at study entry, which increased to a total 7888 patients with a diagnosis of NIDCM at study end. NIDCM patients were older and more often male compared with the general population (median age: 60 years vs 36 years; male proportion: 70% vs 50%, respectively). Furthermore, NIDCM patients also had a higher presence of comorbidities and medication usage compared with the general population (Supplementary Table S4).

### Incidence of out-of-hospital cardiac arrest

**Figure 2:**
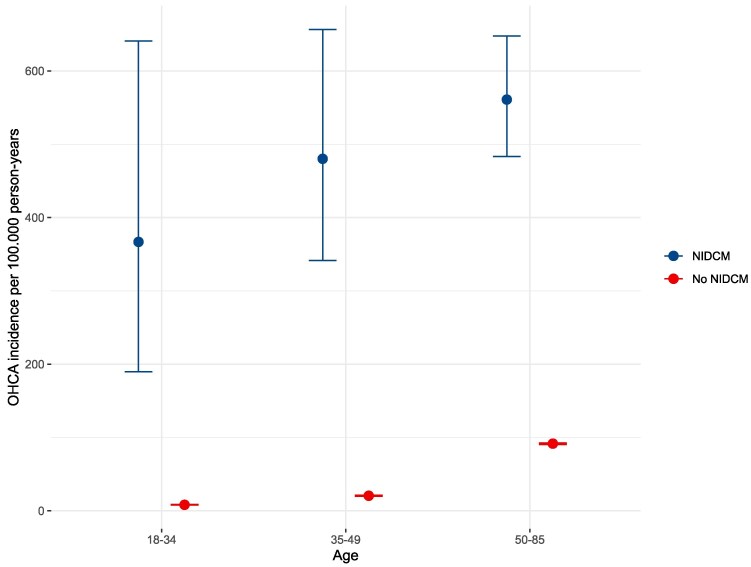
Age stratified incidence rate of out of hospital cardiac arrest. Graph comparing the age-stratified incidence rates for OHCA in NIDCM versus the general population. The figure demonstrates the marked increase in those with NIDCM. Abbreviations: OHCA, out-of-hospital cardiac arrest; NIDCM, non-ischemic dilated cardiomyopathy

During 88.2-million-person years of follow-up we observed 237 OHCAs in persons with NIDCM and 37 116 OHCAs in persons without NIDCM, corresponding to incidence rates of 532 (95%-CI 466–604) and 45 (95%-CI 45–46) per 100 000 person-years respectively and an IRR of 11.79 (95%-CI 10.35–13.36). Male and female OHCA incidence rates per 100 000 person years were 614 (95%-CI 530–708) vs 348 (95%-CI 257–462) in individuals with NIDCM, and 60 (95%-CI 60–61) vs 30 (95%-CI 30–31) in non-NIDCM. When stratified by age groups (18–34, 35–39 and 50–85), OHCA incidence rates were higher across all NIDCM groups compared to non-NIDCM: 367 (95%CI 189–641), 480 (95%-CI 341–656) and 561 (95%-CI 483–648) vs 8 (95%-CI 7.9–8.6), 21 (95%-CI 20–21), and 92 (95%-CI 91–93) respectively (*Figure 2*). The HR for OHCA was higher in NIDCM (HR: 3.48, 95%-CI 3.05–3.96) compared with several other cardiovascular and non-cardiovascular diseases (stroke, atrial fibrillation, and diabetes etc. Cox-model hazard ratios in *Table 1*).

**Table 1:** Hazard ratios of OHCA and mortality in general population

	OHCA	Mortality
Male sex	2.20 (2.16–2.25)	1.38 (1.37–1.39)
NIDCM	3.48 (3.05–3.96)	1.65 (1.56–1.75)
Atrial fibrillation	1.65 (1.58–1.73)	1.74 (1.72–1.76)
Stroke	1.53 (1.47–1.60)	2.72 (2.69–2.75)
COPD	4.00 (3.87–4.13)	3.49 (3.45–3.52)
Peripheral artery disease	2.11 (1.98–2.25)	2.47 (2.43–2.51)
Diabetes	1.65 (1.59–1.72)	1.86 (1.84–1.88)
Cancer	1.73 (1.68–1.78)	8.28 (8.22–8.33)
Statin use	0.92 (0.89–0.94)	0.53 (0.53–0.54)

Hazard ratios with 95% confidence intervals.

COPD, chronic obstructive pulmonary disease; NIDCM, non-ischaemic dilated cardiomyopathy; OHCA, out-of-hospital cardiac arrest.

### Long-term risk of out-of-hospital cardiac arrest

A total of 6718 *de novo* NIDCM patients (median age: 59 [IQR: 49, 68]; males 4689 [70%]) and 26 872 matched controls (median age: 59 [IQR: 49, 68]; males 18 756 [70%]) were included in the exposure matched cohort and used to estimate the long-term risk of OHCA. Baseline characteristics of the exposure-matched cohort are shown in Supplementary Table S3.

**Figure 3:**
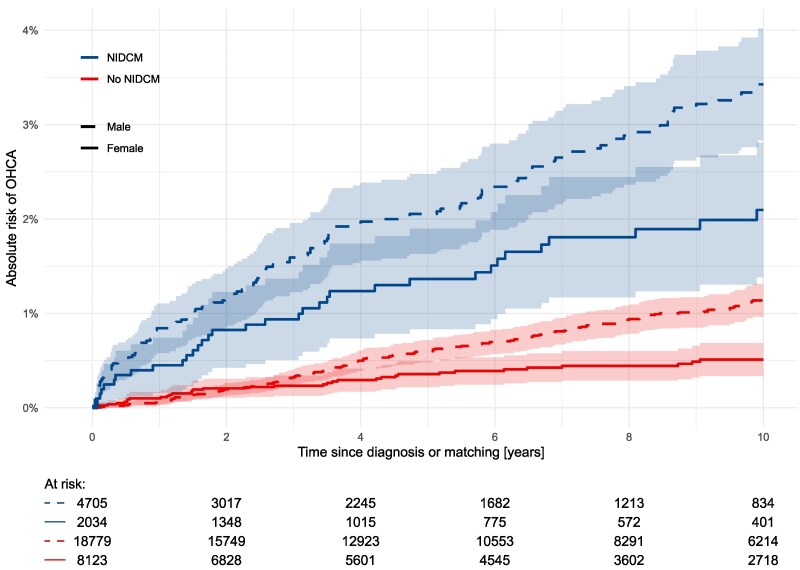
Cumulative incidence of OHCA as time since date of diagnosis or matching in patients with NIDCM and matched controls stratified by sex. Abbreviation: OHCA, out-of-hospital cardiac arrest; NIDCM, non-ischemic dilated cardiomyopathy. The figure clearly identifies male NIDCM patients as being the highest risk cohort

*Figure 3* shows the long-term risk of OHCA stratified by sex. The results showed that the risk of OHCA was highest for male NIDCM patients, with 5- and 10-year risk of OHCA being 2% (95%-CI 1.6–2.5) and 3.4% (95%-CI 2.8–4.0), respectively vs 0.6% (95%-CI 0.5–0.7) and 1.1% (95%-CI 0.9–1.3) for non-NIDCM males. Female NIDCM had a 5- and 10-year risk of 1.4% (95%-CI 0.8–1.9) and 2.1% (95%-CI 1.4–2.8), respectively vs 0.4% (95%-CI 0.2–0.5) and 0.5% (95%-CI 0.3–0.7) for non-NIDCM females.

**Figure 4:**
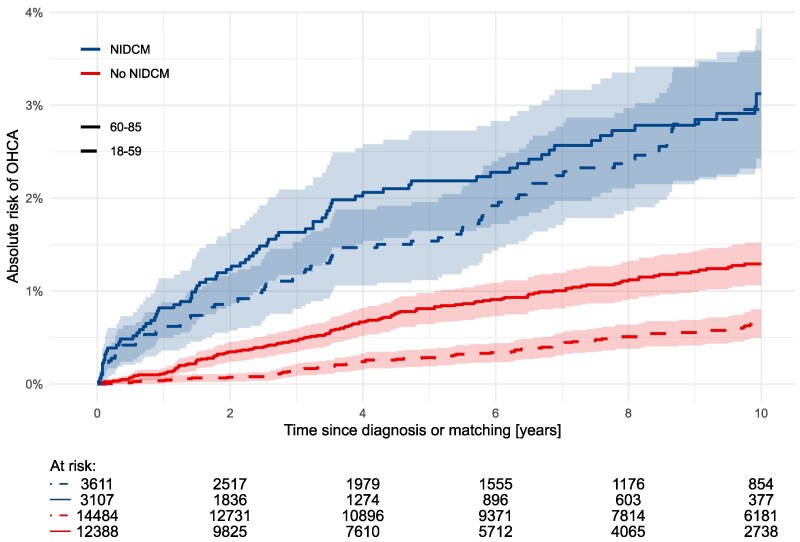
Cumulative incidence of OHCA as time since date of diagnosis or matching in patients with NIDCM and matched controls stratified by age groups. Abbreviations: OHCA, out-of-hospital cardiac arrest; NIDCM, non-ischaemic dilated cardiomyopathy. The figure shows that the risk of OHCA was highest in NIDCM, with no difference between the two age groups compared with the non-NIDCM patients

*Figure 4* shows the long-term risk of OHCA stratified by age groups (18–59 and 60–85). The results show the risk of OHCA was highest in NIDCM, with no difference between the two age groups compared with the non-NIDCM patients, for which the risk of OHCA was higher in the older age group compared with the younger age group. In age group 18–59 years, the 5- and 10-year risk estimates for NIDCM patients were 1.5% (95%-CI 1.1–2.0) and 3.0% (95%-CI 2.3–3.6), and for non-NIDCM patients 0.3% (95%CI 0.2–0.4) and 0.7% (95%CI 0.5–0.8). In age group 60–85 years the 5- and 10-year risk for NIDCM patients were 2.2% (95%-CI 1.6–2.7) and 3.1% (95%-CI 2.4–3.8) and for non-NIDCM 0.8% (95%-CI 0.6, 1.0) and 1.3% (95%-CI 1.1–1.5).

### Characteristics of out-of-hospital cardiac arrest

To investigate the characteristics of OHCA in NIDCM, the 237 NIDCM OHCAs were incidence density matched (sex, age, calendar year) to 936 non-NIDCM-OHCA patients (*Table 2*). NIDCM OHCAs were more often bystander-witnessed (50.2% vs 42.8%) and had more bystander-performed CPR (78.4% vs 68.1%), along with a higher rate of initial shockable rhythm (38.5% vs 22.3%). Non-NIDCM OHCAs had more Emergency Medical Service (EMS) witnessed arrests (10.8% non-NIDCM vs 5.9% NIDCM). 30-Day mortality after OHCA was similar, with no statistical difference between the two groups (79% NIDCM OHCA vs 84% non-NIDCM OHCA, *P* = .3).

**Table 2:** Demographics and characteristics of OHCA in patients with and matched controls without NIDCM

Characteristic	OHCA with NIDCM *N* = 237	OHCA without NIDCM *N* = 936	*P*-value
**Age at OHCA/match (years)**	62.0 (52.0, 72.0)	62.5 (52.0, 72.0)	
**Sex (male)**	189 (79.7%)	749 (80.0%)	
**Year of OHCA/match**	2014 (2008, 2017)	2014 (2008, 2017)	
**Location of OHCA**			.9
Private	134 (72.8%)	528 (71.9%)	
public	50 (27.2%)	206 (28.1%)	
Unknown	53	202	
**Witnessed OHCA**			.028
Bystander	119 (50.2%)	401 (42.8%)	
EMS-personnel	14 (5.9%)	101 (10.8%)	
Unwitnessed	104 (43.9%)	434 (46.4%)	
**CPR, performed**			
Bystander	149 (78.4%)	464 (68.1%)	.008
EMS-personnel	138 (58.2%)	539 (57.6%)	>.9
**DC, performed**			
Bystander	20 (8.5%)	49 (5.2%)	.084
EMS-personnel	108 (45.8%)	299 (31.9%)	<.001
**Initial rhythm**			<.001
Non-shockable	139 (61.5%)	687 (77.7%)	
Shockable	87 (38.5%)	197 (22.3%)	
**Status at hospital arrival**			.068
No ROSC	166 (70.0%)	709 (76.1%)	
ROSC	71 (30.0%)	223 (23.9%)	
**Post OHCA mortality**			
30-day mortality	187 (79.2%)	779 (83.6%)	.14
1-Year mortality	186 (81.6%)	764 (84.1%)	.4

Wilcoxon rank sum test; Pearson's Chi-squared test.

CPR, cardiopulmonary resuscitation; DC, direct cardioversion; EMS, emergency medical services; ROSC, return of spontaneous circulation.

## Discussion

This nationwide register-based study showed that the incidence rate of OHCA in patients with NIDCM was approximately 12 times greater than the incidence rate in the general population. In addition, after adjusting for covariates, the hazard ratio of OHCA remained significantly increased for NIDCM (HR: 3.48, 95% [3.05–3.96]). Furthermore, risk analysis in the exposure-matched cohort showed an increased long-term risk of OHCA in both male and female NIDCM patients compared with matched controls (male: 3.4% vs 1.1%; female: 2.1% vs 0.5%, respectively). Additionally, despite having more positive resuscitation characteristics, NIDCM patients had similar survival after OHCA compared to the non-NIDCM cohort. However, despite this increased incidence and risk, the absolute event rate and annual risk of OHCA in the NIDCM cohort remains low.

### Identifying those at-risk for SCD

The NIDCM population is a heterogenous cohort with varying aetiologies, phenotypes, and SCD risk. As such, obtaining data to develop risk stratification methods for such a variable population remains challenging. Left ventricular ejection fraction (LVEF) of <35% has historically been key to both SCD risk stratification and primary prevention implantable cardioverter defibrillator (ICD) eligibility in the NIDCM population, but its recent guideline demotion from a class 1A indication for primary prevention ICD implantation to class 2A^3,4^ reflects the growing dominance of multi-parametric risk stratification. Progress^18^ in other modalities is being made however, namely in imaging, with the presence and extent of late gadolinium enhancement (LGE) on cardiac magnetic resonance imaging^19^ gradually overtaking LVEF as the primary predictor of SCD risk in NIDCM.^20^ Conduction abnormalities (microvolt T-wave alternans, 1st degree AV block,^21^ periodic repolarization dynamics^22^) and genetics (the validated risk score for patients with a confirmed Lamin-A mutation^23^) allow further risk stratification, whilst a recent study^24^ provided temporal risk data with the identification of a ‘vulnerability phase’ for NIDCM patients in the first 200 days from diagnosis, where VA and subsequent SCD incidence was highest.

### Preventing SCD in non-ischaemic cardiomyopathy

Current medical treatment for NIDCM focuses on halting the progression of systolic dysfunction with rapid commencement on guideline-directed medical therapy (GDMT): beta blockers, angiotensin receptor/neprilysin inhibitors (ARNIs), mineralocorticoid receptor antagonists (MRAs) and sodium-glucose co-transporter 2 inhibitors (SGLT2i). However, up to 35% of NIDCM mortality is due to SCD as opposed to progressive systolic dysfunction, and a significant proportion of patients with well-controlled heart failure symptoms (NYHA Class <II) will experience SCD^25^ (often preceded by an OHCA).

The immediate detection and termination of a VA that leads to SCD relies on the presence of an initial shockable rhythm (one of the strongest predictors of OHCA survival^26^) and means of early defibrillation, primarily in the form of an ICD. However, ICD utility in the NIDCM cohort is under scrutiny, with the pivotal trials comparing differing combinations of the optimal medical therapy (OMT) of the time with ICD’s offering variable results. DEFINITE^27^ (2004) randomized patients with NIDCM and LVEF <35% to either ICD and OMT—primarily beta blockers and angiotensin-converting enzyme inhibitors (ACEi)—or OMT alone and found a reduction in SCD but no reduction in all-cause mortality. Alternatively, SCD-HeFT^28^ (2005) demonstrated reduced all-cause mortality in a mixed ischaemic and non-ischaemic cohort, which tracked into the non-ischaemic cohort subgroup analysis (albeit limited power impaired a statistically significant result). The publication of DANISH^29^ (2016), and its follow ups,^30^ more recently examined ICD efficacy in the non-ischaemic cohort, demonstrating a reduction in SCD in those aged <70 but once again no effect on all-cause mortality. These trials remain difficult to interpret due to their emergence over a period of significant development in heart failure pharmacotherapy, namely the emergence of modern GDMT, but before the widespread uptake of ANRIs and SGLT2i. Evidence that GDMT reduces the risk of SCD exists^31^ but opinions remain divided.^32^

Our analysis of resuscitation characteristics demonstrated a significantly higher rate of initial shockable rhythm in NIDCM OHCA vs non-NIDCM OHCA (38.5% vs 22.3%, *P* ≤ .001). Although there is a signal to suggest a proportion of the NIDCM OHCA patients may have benefitted from an ICD (including those with non-shockable rhythms which may have been initially shockable but subsequently degenerated to asystole by the time of rhythm analysis), the data does not allow us to draw further conclusions due to a lack of clinical parameters such as LVEF, NYHA class, and imaging/LGE. Furthermore, analysis of 5 and 10-year risk of OHCA showed persistent but modest risk increases across all groups, with the highest risk demographic (male NIDCM) remaining only 3.4% at 10 years. When stratified by age, risk was highest in NIDCM patients aged 60–85 at 10 years (3.1%) but did not differ significantly from the younger cohort 18–59 (3%). Clinically, these 5- and 10-year OHCA analyses provide tangible data to both patients and clinicians regarding long-term risk, which can inform discussions about interventions and therapy. Importantly, the low absolute risk (and event rate) at 10 years is particularly relevant for NIDCM patients with low LGE burden, minimal symptoms or mild-to-moderately reduced LVEF, and may promote more conservative and individualized decision-making for primary prevention ICD implantation—an intervention which carries a number of peri-procedural and life-long risks including infection, lead malfunction, and inappropriate shocks.

### Survival after OHCA

OHCA continues to have an extremely poor prognosis globally, despite major advances in both pre-hospital resuscitation and post-arrest care for those who obtain return of spontaneous circulation (ROSC). Alongside initial shockable rhythm, OHCA survival in all cohorts is influenced by the interaction of multiple factors including underlying cause of arrest, early CPR, rapid defibrillation and avoidance of post-cardiac arrest complications^33^ (namely ischaemia-reperfusion syndrome [IRS], post-arrest myocardial dysfunction [PAMD] and hypoxic neurologic injury).^34^

We analysed several arrest and resuscitation characteristics including location, CPR and delivery of defibrillation. Location of cardiac arrest (public vs private) was similar across both groups; however, the NIDCM cohort received a higher rate of bystander CPR (78.4% vs 68.1%). The higher rate of EMS-witnessed OHCAs in the non-NIDCM cohort may reflect the higher rate of CAD in the general population, who likely experience a symptomatic prodrome (such as ischaemic chest pain) prompting EMS contact prior to their arrest as opposed to the more rapid onset of NIDCM arrests (due to sudden VA). Furthermore, despite less EMS-witnessed OHCAs, NIDCM OHCAs had a higher rate of EMS-direct defibrillation (EMS-DC) (46% vs 32%), likely because of the higher rate of initial shockable rhythm.

However, despite the higher rate of initial shockable rhythm, bystander CPR and EMS-DC in the NIDCM cohort, there was no difference in 30-day and 1-year mortality after OHCA compared to the non-NIDCM group—an unexpected finding given the clear resuscitation advantages described in the NIDCM cohort. Possible explanations may lie in the underlying pathophysiology of NIDCM, which likely blunts the effect of positive resuscitation characteristics, especially when combined with post-cardiac arrest complications. Myocardial recovery may be impaired by the underlying structural abnormalities of NIDCM and further exacerbated by the development of PAMD, leading to persistently impaired cardiac function and worse neurologic injury (the primary cause of death post-OHCA resuscitation.^35^) Additionally, the increased presence of interstitial fibrosis characteristic of NIDCM may act as a substrate for refractory arrhythmias.^36^ Furthermore, non-arrhythmic causes of OHCA may not necessarily benefit from the advantage of an initial shockable rhythm or other positive resuscitation characteristics. Alternatively, the comparable mortality rate may be secondary to the higher prevalence of positive resuscitation characteristics, which bring the mortality of a cohort with known structurally abnormal hearts in line with that of a (presumed) structurally normal cohort.

### Identifying the correct study population within the limitations of the registries

Nationwide register-based studies offer several benefits including large sample sizes, long observation periods, and increased statistical power. However, identifying an accurate study population, in this case a truly ‘*non-ischaemic*’ DCM cohort, using population-level registries is subject to numerous challenges and limitations. To mitigate these, we first used the previously validated ICD-10 code for non-ischaemic aetiology of DCM, accurate in up to 82% of cases (although we acknowledge earlier NIDCM cases may not correspond completely with most recent ESC guideline definitions.^1^) To refine this further, we excluded any individuals with additional pathologies that could promote ischaemia or abnormal loading conditions—what we term ‘explainable diseases’. This resulted in a study population within the limits of what registries will allow.

## Limitations

Observational register studies are subject to numerous limitations. Firstly, although the ICD code used is specific and validated for non-ischaemic DCM, it may be inaccurate in up to 18% of cases (although steps were taken to attenuate this with further exclusion of explained causes of DCM in our study population). Secondly, nationwide register-based studies rely on correct entry of data and as such are always subject to human or administrative error, misclassification or omission. Finally, absence of key patient-level data such as LVEF, familial/genetic history, imaging data such as LGE and, more specifically for SCD, the presence of undetected coronary artery disease, coupled with long observation period and the low frequency of NIDCM OHCA events, limit the conclusions that can be drawn from our findings.

## Conclusion

The NIDCM population has a significantly increased risk of OHCA compared to the general population. However, even for the highest risk cohort, 5- and 10-year OHCA incidence remained low. Additionally, although the NIDCM OHCA cohort had a higher number of positive resuscitation and arrest characteristics, there was no difference in mortality compared to the non-NIDCM OHCA population.

## Supplementary Material

xvag207_Supplementary_Data

## Declarations

### Data Availability

No data were generated or analysed for this manuscript.

### Pre-registered Clinical Trial Number

None supplied.
