# Supplementary material for: Incidence, analysis and risk of out-of-hospital cardiac arrest in individuals with non-ischaemic dilated cardiomyopathy
Source: ESC Heart Fail. 2026 Jul 31;13(4):xvag207. doi: 10.1093/eschf/xvag207 (PMC13475126; doi:10.1093/eschf/xvag207)
Supplement: Supplementary file 1 [file xvag207_supplementary_data.zip › Supplementary Appendix - Revised.docx]

**Supplementary Appendix**

**Content**

[Supplementary Table 1. List of ICD 8 & 10 Codes used to Identify Explainable Diseases	2](#_Toc230850341)

[Supplementary Table 2. List of ATC Codes used to Identify Hypertension	2](#_Toc230850342)

[Supplementary Table 3. Cohort Study Baseline Characteristics of NIDCM-Patients and Matched General Population to Determine 5- and 10-Year Risk of OHCA	3](#_Toc230850343)

[Supplementary Table 4. Clinical and demographic characteristics at baseline and according to NIDCM status	4](#_Toc230850344)

[Supplementary Figure 1. Absolute risk of mortality as first event and development of explainable disease in NIDCM patients and matched general population	5](#_Toc230850345)

| **Supplementary Table 1.** List of ICD 8 & 10 Codes used to Identify Explainable Diseases | | |
| --- | --- | --- |
| Disease | **ICD 8 codes** | **ICD 10 codes** |
| Ischemic Heart Disease | 41009, 41099, 41109, 41209, 41299, 41309, 41399, 41409, 41499 | DI20, DI21, DI22, DI23, DI23, DI25, |
| Chronic Kidney Disease | 24902, 25002, 58200, 58201, 58202, 58208, 58209, 58300, 58301, 58302, 58309, 58499, 59009, 59320, 75310, 75311, 75319, 79299, 40399, 40499, 44609, 44629 | DN02, DN03, DN04, DN05, DN06, DN07, DN08, DN11, DN12, DN14, DN18, DN19, DN26, DN158, DN159, DN160, DN162, DN163, DN164, DN168, DQ61, DE102, DE112, DE132, DE142, DI120, DM312B, DQ612, DQ613. DQ615, DQ619 |
| Valve Disease | None | DI34, DI35 |
| Abbreviation: ICD 8/10- International Classification of Diseases 8th/10^th^ Revision | | |

| Supplementary Table 2. List of ATC Codes used to Identify Hypertension | |
| --- | --- |
| Medication | **ATC-codes** |
| Anti Adrenergic | C02A, C02B, C02C |
| Diuretics | C02DA, C03A, C03B, C03D, C03E, C03X, C02DA, C07B, C07C, C07D, C08G, C09BA, C09DA, C09XA52, C02L |
| Vasodilators | C02DB, C02DD, C02DG |
| Beta blockers | C07A, C07B, C07C, C07D, C07F |
| Calcium Channel Antagonist | C07FB, C08, C08G, C09BB, C09DB |
| Renin-Angiotensin System Inhibitors | C09AA, C09BA, C09BB, C09CA, C09DA, C09DB, C09XA02, C09XA52 |
| Abbreviations: ATC = Anatomical Therapeutic Chemical | |

| **Supplementary Table 3.** Cohort Study Baseline Characteristics of NIDCM-Patients and Matched General Population to Determine 5- and 10-Year Risk of OHCA | | | |
| --- | --- | --- | --- |
| **Characteristic** | **No NIDCM** N = 26,872^1^ | **NIDCM**  N = 6,718^1^ | **p-value**^2^ |
| Age (years) | 59 (49, 68) | 59 (49, 68) | - |
| Sex (male) | 18,756 (70%) | 4,689 (70%) | - |
| **Comorbidities** | | | |
| Stroke | 1,244 (4.6%) | 311 (4.6%) | - |
| Atrial fibrillation | 5,040 (19%) | 1,260 (19%) | - |
| Chronic obstructive pulmonary disease | 2,088 (7.8%) | 522 (7.8%) | - |
| Peripheral artery disease | 304 (1.1%) | 81 (1.2%) | 0.6 |
| Cancer | 2,520 (9.4%) | 630 (9.4%) | - |
| Diabetes | 2,388 (8.9%) | 597 (8.9%) | - |
| **Medication** | | | |
| Statins | 5,660 (21%) | 1,415 (21%) | - |
| Diuretics | 5,580 (21%) | 2,571 (38%) | <0.001 |
| Betablocker | 5,692 (21%) | 3,137 (47%) | <0.001 |
| Calcium channel antagonist | 3,961 (15%) | 830 (12%) | <0.001 |
| Renin-angiotensin inhibitor | 5,239 (19%) | 3,476 (52%) | <0.001 |
| ADP inhibitor | 463 (1.7%) | 134 (2.0%) | 0.13 |
| **First event** |  |  |  |
| Censored | 18,004 (67%) | 2,886 (43%) |  |
| OHCA | 263 (1.0%) | 185 (2.8%) |  |
| Mortality | 2,653 (9.9%) | 939 (14%) |  |
| Development of any explainable disease | 5,952 (22%) | 2,708 (40%) |  |
| Abbreviations: ADP = adenosine diphosphate; NIDCM = non-ischemic dilated cardiomyopathy; OHCA = out-of-hospital cardiac arrest  ^1^Median (Q1, Q3); n (%) | | | |
| ^2^Wilcoxon rank sum test; Pearson's Chi-squared test | | | |

| **Supplementary Table 4.** Clinical and demographic characteristics at baseline and according to NIDCM status | | | | |
| --- | --- | --- | --- | --- |
| **Characteristics** | **At Start of Observation** |  | **At Last Covariate Update Before Event or Censoring** | |
|  | **Whole population**  N = 6,194,082 |  | **NIDCM** N = 7,888 | **No NIDCM** N = 6,186,194 |
| Age (Years) | 32 (19, 50) |  | 60 (50, 70) | 36 (21, 59) |
| Sex (Male) | 3,081,141 (50%) |  | 5,515 (70%) | 3,075,614 (50%) |
| **Comorbidities** | | | | |
| NIDCM | 1,047 (<0.1%) |  | - | - |
| Atrial Fibrillation | 26,740 (0.4%) |  | 2,788 (35%) | 170,229 (2.8%) |
| Stroke | 47,807 (0.8%) |  | 662 (8.4%) | 191,651 (3.1%) |
| Peripheral Artery Disease | 20,435 (0.3%) |  | 208 (2.6%) | 52,883 (0.9%) |
| Chronic Obstructive Pulmonary Disease | 49,628 (0.8%) |  | 993 (13%) | 183,807 (3.0%) |
| Cancer | 122,191 (2.0%) |  | 1,254 (16%) | 586,910 (9.5%) |
| Hypertension | 127,420 (2.1%) |  | 4,488 (57%) | 446,640 (7.2%) |
| Diabetes | 77,244 (1.2%) |  | 1,819 (23%) | 395,521 (6.4%) |
| **Medication** |  |  |  |  |
| Diuretics | 347,486 (5.6%) |  | 5,676 (72%) | 1,097,316 (18%) |
| Beta Blocker | 242,224 (3.9%) |  | 6,839 (87%) | 818,852 (13%) |
| Calcium Channel Antagonist | 145,504 (2.3%) |  | 1,555 (20%) | 765,852 (12%) |
| Renin-Angiotensin System Inhibitor | 155,993 (2.5%) |  | 7,312 (93%) | 1,104,565 (18%) |
| Mineralocorticoid Receptor Antagonist | 18,977 (0.3%) |  | 4,798 (61%) | 118,861 (1.9%) |
| Statins | 38,380 (0.6%) |  | 3,451 (44%) | 877,106 (14%) |
| Adenosine Diphosphate Receptor Inhibitors | 858 (<0.1%) |  | 376 (4.8%) | 115,691 (1.9%) |
| Data is presented as median (Q1, Q3) and n (%)  Abbreviations: NIDCM = non-ischemic dilated cardiomyopathy. | | | | |

# **Supplementary Figure 1.** Absolute risk of mortality as first event and development of explainable disease in NIDCM patients and matched general population
